# Some Experiments on the Action of Organic Matters upon Silver Salts

**Published:** 1882-06

**Authors:** Henry Leffmann


### SOME EXPERIMENTS ON THE ACTION OF ORGANIC MATTERS UPON SILVER SALTS.

---

The action of organic matter upon silver salts is well known, but I am not aware of any attempts to utilize this method for the examination of water. The following experiments were undertaken as a sort of preliminary investigation. The subject of water analysis is so important, and so much remains to be done, that every observation of the kind must have some value.

If we add a salt of silver to ordinary water, the precipitated chloride interferes with the test, and to prevent this I used a solution containing marked excess of ammonia. In the following experiments, the proportion used was 2 *cc.* of ammonio-nitrate of silver to 100 *cc.* of the water.

This silver solution contained only a few grains to the ounce. When not otherwise mentioned, the water was exposed to the sunlight for two hours.

1. Distilled water . . . . . . . . . . . . . . . . . . . . . . . . . . . . No color.
2. Schuylkill water . . . . . . . . . . . . . . . . . . . . . . . . . . . . No color.
3. Schuylkill water with 0.1 *cc* urine . . . . . . . . . . . . . . . . . Brown color.
4. Schuylkill water with 0.5 *cc.* urine . . . . . . . . . . . . . . . . . Deep brown.
5. Schuylkill water with 0.02 *cc.* urine . . . . . . . . . . . . . . . . Red-brown.
6. Schuylkill water with 4 grs. raw sugar . . . . . . . . . . . . . . . No color.
7. Schuylkill water with 2 grs. stale mash . . . . . . . . . . . . . . . Yellowish.
8. Well water, not perfectly pure, but not unfit to drink . . Faint black.
9. Well water, markedly contaminated
   Black precipitate almost immediately.
10. Water from a small stream, quite pure . . . . . . . . . . . . . . . No color.

Waters containing small amounts of milk, glucose, and albumen gave no distinct effects. Solution of glue produced a faint brown. All the experiments tended to show that the test was very sensitive to the presence of urine. Some experiments were made with highly dilute solutions of the common active principles.

Quinidia, strychnia, and cinchonidia gave no result. Picrotoxin gave light yellow; caffeine gave light yellow; quinidine sulphate gave faint brown; morphia gave immediate precipitate.

I hope to present before long the results of some further study of the matter.—*Dr. Henry Leffmann in The Analyst.*

